# Contribution of Grape Juice to Develop New Isotonic Drinks With Antioxidant Capacity and Interesting Sensory Properties

**DOI:** 10.3389/fnut.2022.890640

**Published:** 2022-06-07

**Authors:** Yasmina Bendaali, Cristian Vaquero, Carmen González, Antonio Morata

**Affiliations:** Department of Chemistry and Food Technology, Escuela Técnica Superior de Ingenieria Agronomica, Alimentaria Y de Biosistemas (ETSIAAB), Universidad Politécnica de Madrid, Madrid, Spain

**Keywords:** grape juice, sport, minerals, carbohydrates, polyphenols, isotonic

## Abstract

Nowadays, the sector of isotonic beverages has developed its market based on fruit juices that provide a sports drink with antioxidant and biological activities in addition to their principal role of rehydration and replacement of minerals and carbohydrates during physical exercise. Consumption of grape juice is increasing worldwide because of its sensory characteristics and nutritional value. It contains mainly water, sugars, organic acids, and phenolic compounds. Phenolic compounds play a major role in prevention of various diseases through their biological activities linked to antioxidant, anti-inflammation, anticancer, anti-aging, antimicrobial, and cardioprotective properties. Several studies have demonstrated that grape juice is able to improve performances of antioxidant activity, protect against oxidative damage, and reduce inflammation during sports activities. Polyphenol content also provides a great sensory profile, mainly color which is an important indicator for consumers when choosing beverage products. The contribution of grape juice through its nutritional value and sensory properties makes it an alternative for the development of a new isotonic drink that will be a novel and healthy product in the field of healthy beverages.

## Graphical Abstract

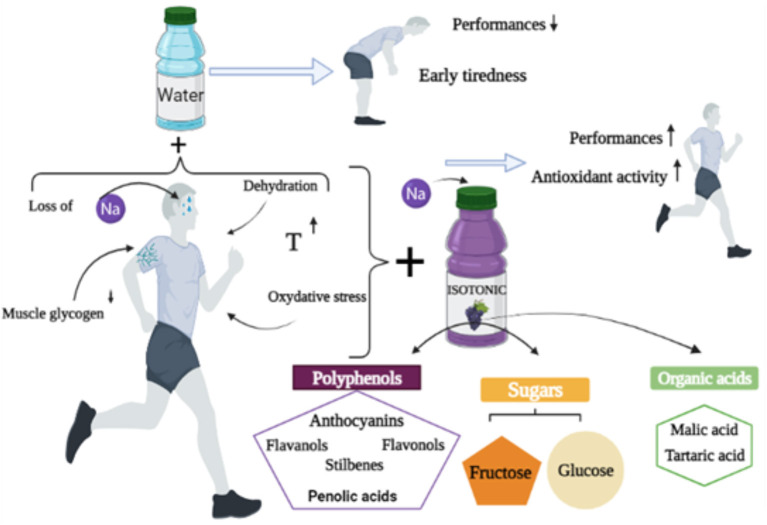

## Introduction

Consumption of fruits and their juices plays an important role in a healthy diet (1) because they are a source of free sugars and micronutrients (2). Even more, consumer tendencies to healthy eating habits (3) and request for organic foods (4) led to the production of new drinks from fruit juices as a source of nutrients and bioactive compounds (5). Nowadays, among the challenges facing the food industry are expansion in the creation of new products and persuading consumers to buy them. In this case, it is essential to recognize the needs and consumers' demands while developing new and innovative products (6).

Several studies focused on the development of functional and healthy beverages based on fruit juice; among them is our previous study (7), where we elaborated on a natural beverage based on red grape juice as a source of polyphenols and sugars, and has interesting organoleptic properties without chemical additives. Another study by Shams Najafabadi et al. (8) in which a beverage was developed based on jujube fruit which is well known for its content in anthocyanins, organic acids, and phenolic compounds. A mixed fruit beverage was developed through sensory analysis by Bhalerao et al. (9) using pomegranate, amla and muskmelon juice (1). formulated and studied the nutritional, organoleptic, physicochemical, phytochemical, microbiological and shelf-life of a natural beverage formulated from orange (*citrus sinensis*), lemon (*citrus limon*), honey, and ginger (*zingiber officinale*). New beverages were also created by the team (10) based on lemon juice with elderberry and grape concentrates as a source of bioactive compounds. Accordingly, the sector of isotonic beverage has developed its market based on fruit juices (11, 12). Isotonic drinks are a group of functional beverages defined as products with beneficial effects on human health in addition to their nutritional effect (6). Furthermore, from the available recent studies related to isotonic drinks, researchers focused their interest in the development of isotonic beverages using a fruit juice with antioxidant and biological activities. An isotonic beverage with functional attributes and as an innovative proposal was developed by Porfírio et al. (12) based on extracts of peels and pulp of *Myrciaria jaboticaba*, which is considered as a rich source of anthocyanins. In another study by the group of (13), the phenol extract of jabuticaba peels with whey ultrafiltration permeate was used in the development of an isotonic beverage (14) designed a new isotonic drink with high antioxidant capacity by mixed berries (maqui, açai and blackthorn) and lemon juices.

Given this context, the objective of this review is to provide an overview of the various characteristics of isotonic beverages, the importance of their mineral and carbohydrate contents, and the contribution of fruit juices in improving nutritional value and sensory profile concerning an isotonic beverage based on grape juice with good organoleptic properties.

## Isotonic Beverage

Loss of water and electrolytes and diminution of glycogen in the liver and muscles owing to increase in metabolic rate and increased heat during physical activity may cause dehydration, which influences physical performance (15) and can lead to early tiredness, cognitive changes, sodium deficit, and increases risk of heat-related diseases (16). Hydration improves thermoregulation, assures the preservation of physiological functions, and helps in suitable functioning of homeostatic mechanisms needed when exercising (17). Generally, water is the first option for hydration before, during, and after sport activities (16). It has been documented that ingestion of water for rehydration can provoke hyponatremia, and that its ingredients are insufficient to provide energy (18). In addition, intake of a high amount of water to replace fluid loss causes a feeling of fullness, urination, decrease in plasma sodium, and early ending of exercise (19). Accordingly, it was mentioned that the best-attained hydration was with a mixture of water, carbohydrates, and electrolytes (20). In addition, consumers consider sports drinks more beneficial than water in terms of electrolytes, minerals, and physical performance (21).

Sports drinks are beverages used before, during, and after physical exercises to replace water, electrolytes, and carbohydrates lost during exercise (22, 23) in order to keep hydration and delay fatigue (24). They can be hypertonic containing a higher concentration of sugar and salt than those which are found in the human body, isotonic containing similar concentrations of sugar and salt as in the human body, or hypotonic containing lower concentrations of sugar and salt than those in the human body (23, 25). For adequate hydration, drinks must be isotonic (26). Intake of isotonic drinks for intensive physical training is recommended, while for extremely intensive training, hypertonic drinks are recommended to speed up the restoration of energy reserves by their easily digestible carbohydrate content (27). Water, carbohydrates, and electrolytes are the main components of isotonic drinks (14, 28). They may also contain vitamins, colors, flavors, and natural juices that improve the organoleptic characteristics (29), and have a pH value close to 3 (30). Taste, acidity, sweetness, and beverage temperature are features that made isotonic beverages more consumable than water during sport activities (11).

### Mineral Content

In current production, isotonic beverages consist of a natural or artificial source of electrolyte salts (29), mainly ions of sodium, potassium, magnesium, chloride, and calcium (31). Sodium is the main cation in the extracellular space, and a large quantity of this cation can be lost by sweating (32). Salt is added to sports beverages in order to provide sufficient quantities of sodium and keep up its concentration and the volume of plasma (33). Sodium plays a major role in the adjustment of the balance of water in the body and transport of carbohydrates and proteins to tissues (34), increases the intestinal absorption of carbohydrates (19, 35), aids the active working muscle to contract and relax (36), and prevents hyponatraemia (28), which is the lack of sodium in the body caused by loss of sodium due to sweating, diarrhea, and vomiting (34).

Potassium creates and preserves constant muscle contraction and nerve impulse, prevents clotting of blood and keeps its pH level, helps in the storage of carbohydrates in the muscles (36), and sets the intracellular water content by its intervention in the synthetic processes of proteins and carbohydrates (26).

Chloride plays an important role in the safety of osmotic pressure and acid-base balance of the body and is necessary for the gastric juice (26, 36).

According to (28), isotonic drinks should contain 0.5–0.7 g/L of sodium and 6–9% carbohydrates, and the concentration of sodium should be increased to 0.7–1.2 g/L during an exercise in the heat and for more than 1 h. However, (34) obtained a level of 342 mg/L sodium in isotonic drinks. In another study by (25), the results of the mineral analysis of a commercial isotonic drink showed a sodium concentration of 45.753 mg/100 ml, potassium 7.526 mg/100 ml, calcium 0.327 mg/100 ml, and magnesium 0.326 mg/100 ml.

### Carbohydrate Content

During intense exercise, muscle glycogen stores and blood glucose decrease which requires a continuous source of carbohydrates to provide energy and avoid fatigue. Isotonic drinks are composed of carbohydrates as a source of energy in the form of mono- and polysaccharides (glucose, fructose, and maltodextrin) (29) with a recommended concentration of 6–9 % (28). According to (37), the intake of carbohydrates before exercise slows down and prevents homeostatic disturbances, gives an adequate plasmatic volume from the start of the exercise, and contributes to offset the loss of carbohydrate stores, enhancing performances during exercises (35).

We have given in Table 1 the main composition of some isotonic, energy, and fruit juices available in the market in order to give information on the composition of isotonic drinks and compare their compositions with other types of beverages. It is clearly observed that there is a difference between the three groups of beverages. The concentration of carbohydrates and salt differs among the six isotonic drinks. carbohydrate concentrations in isotonic drinks (4.4 ± 0.78 g/100 ml) are lower than the concentrations present in energy drinks and fruit juices. They are ranging from 2.9 to 5 g/100 ml in isotonic drinks, from 9.9 to 17 g/100 ml in fruit juices, and from 11 to 16 g/100 ml in energy drinks. Caloric values are also higher in energy drinks and fruit juices compared to isotonic drinks. Organic grape juice has high carbohydrate concentration among fruit juices (17 g/100 ml). Thus, in our previous study (7), to obtain a healthy beverage from grape juice, we diluted the sugar content to 40–50 g/L with mineral water, because it cannot be considered a healthy beverage if it contains a high concentration of sugars. Therefore, a significant way to improve the impact of grape isotonic juices on health is dilution of sugars to 40–50 g/L with mineral water, which also improves the glycemic index. On the other hand, research shows that simple carbohydrates (sucrose, glucose, and fructose) are most effective in stimulating fast absorption and promoting carbohydrate oxidation. Thus, the amount and type of carbohydrates used in a sports drink are important to optimize the potential of the drink and improve performance (37). In addition, it was mentioned that consumption of carbohydrates with a low glycemic index (GI) before physical exercises has a beneficial effect, because they perform a slow release of glucose into the blood after digestion, providing a sustained source of energy to contracting muscles, maintaining muscle glycogen, and improving performances. They also promote a low insulin response that is beneficial for substrate metabolism, because a high level of insulin inhibits fat lipolysis and oxidation (38). Also, a comparison of sugar, salt, and calorie values in the same group (isotonic, energy, or fruit beverages) demonstrates that there is not a large difference between isotonic beverages composition unlike in the group of energy drinks and fruit juices. However, salt concentrations are higher in isotonic drinks (0.08 ± 0.02 g/100 ml) and absent in fruit juices. Proteins, fats, and fibers are missing in both isotonic and energy drinks, and they are present in fruit juices with a mean value 0.5 ± 0.16 g/100 ml. Stimulants (caffeine and guarana) are found only in energy drinks.

**Table 1:** Composition of isotonic drinks, energy drinks, and fruit juices collected from the label, and available in 100 ml.

**Beverage name**	**Beverage type**	**Carbohydrates** **(g)**	**Salts** **(g)**	**Calories** **(KJ)**	**Proteins** **(g)**	**Fat** **(g)**	**Fibers** **(g)**	**Stimulants**
Aquarius	Isotonic drinks	4.4	0.05	80	–	–	–	–
Powerade		5	0.13	93	–	–	–	–
Isofresh		4.9	0.08	83	–	–	–	–
Raw		2.9	0.105	54	–	–	–	–
Iso on		4.8	0.07	85	–	–	–	–
Isodrink		4.4	0.06	81	–	–	–	–
Mean values		**4.4** **±0.78**	**0.08** **±0.02**	**79.33** **±13.25**	–	–	–	–
Red bull	Energy drinks	11	0.1	195	–	–	–	Caffeine
Coca-cola energy		10.4	0	177	–	–	–	Caffeine Guarana
Burn		16	0.05	263	–	–	–	Caffeine Guarana
Mean values		**12.47** **±3.07**	**0.05** **±0.05**	**211.67** **±45.36**	–	–	–	–
Organic grape juice	Fruits juice	17	–	298	<0.5	–	–	–
Peach juice		10.7	–	187	0.3	–	–	–
Natural orange juice		9.9	–	187	0.7	0.1	0.6	
Grape and peach juice		12.8	–	234	0.5	0.2	–	–
Mean values		**12.6** **±3.18**	**–**	**226.5** **±52.56**	**0.5** **±0.16**	**0.075** **±0.10**	**0.6**	**–**

*Bold values indicate main values are the average ± deviation standard*.

Accordingly, optimization of carbohydrates and salt concentration for performance is an interest of sport nutritionists and drink manufacturers (39). Higher concentration provides more carbohydrate and salt but minimizes the rate of gastric emptying and can consequently delay the rate of delivery of fluids (19, 35). The needed osmolality value (270–330 mOsm/kg of water) (29) defined by the European Food Safety Authority (EFSA) for isotonic drinks (6) which is the same osmolality found in the human body, leads to prevention of tiredness and increase performances after ingestion of the beverage (12) because of fast absorption of water and ions, which is the main purpose of isotonic drink consumption, to replenish the fluids lost during physical exercises (31).

## Contribution of Grape Juice to Isotonic Drinks

### Composition of Grape Juice

New dietary guidelines and health professionals are interested in developing foods with lower sugar content or with alternative sweetener sources due to the multiple diseases associated with sugar intake such as obesity, diabetes, cardiovascular disorders, and cholesterol (40). Zhang et al. (33) mentioned that grape juice sports drinks do not need to use sweeteners and acidulants. Grape juice was chosen for designing an isotonic beverage according to its known bioactivity and nutritional composition. Grape juice is a beverage extracted from different grape varieties, mainly the *Vitis vinifera, Vitis labrusca*, and *Vitis rotundifolia* species (41), by different technological processes (hot press, cold press, and hot break) (42). Its consumption is increasing worldwide, because of its sensory characteristics and nutritional value (43). The United States, Spain, China, Italy, France, Turkey, and Chile are the top producers of grape juice (41). Grape juice contains water, high concentration of sugars and organic acids (42, 44, 45), and minerals, phenolic compounds, and other nutrients such as vitamins, proteins, fatty acids, and amino acids (46, 47). Carbohydrates are found in the form of fructose and glucose (48). The main organic acids in grape juice are tartaric, malic, and citric acids (41), In addition, these acids are used as indicators of microbiological alterations in the beverage because of their impact on its stability (49). Phenolic compounds are the most abundant compounds followed by sugars and acids (41). They play a major role in prevention of various diseases caused by oxidative stress (50). The phenolic compounds found in grape juice are those extracted from grape skins and seeds (42). They are classified into flavonoids such as flavanols, flavonols, and anthocyanins, and non-flavonoids mainly phenolic acids and stilbene (49, 51). Numerous studies have identified and quantified the phenolic content of different varieties and cultivars of grape juice (46, 49, 52–55). The phenolic content differed among various grape juices. Thus, researchers have indicated that the content and profile of phenolic compounds are dependent on grape varieties, species, (43) technology of juice preparation (49), geographical origin, ripeness, type of soil, sunlight exposure, method used for quantification (41), farming system of grapes (organic, conventional, and biodynamic) (56), and culture conditions. Grape tissues in the pulp are rich in phenolic acids, and the skin is rich in flavonoids (57). However, anthocyanins are the main phenolic compounds in red grape juices, while flavan-3-ols are more abundant in white grape juices (42). It is mentioned that most phenolic compounds in white grapes belong to the non-flavonoid group, including mainly phenolic acids such as gallic, vanillic, syringic, protocatechuic, and ellagic acids, flavonoids such as flavanols mainly catechin, epicatechin, procyanidins, and flavonols mainly quercetin and other aglycones (44).

### Health Benefit of Grape Juice

The interest of consumers and the food industry in polyphenols has been growing, because there is a relationship between their intake and prevention of various diseases (50, 54). Accordingly biological activities of polyphenols in grape juice are linked to their antioxidant, anti-inflammation, anticancer, anti-aging, antimicrobial, and cardioprotective properties (58). They can prevent platelet aggregation, LDL, DNA (59), lipid, protein (53), and membrane damage oxidation (57), reduce adhesion molecule expression and limit inflammations (60), which block cellular events predisposing atherosclerosis (61), enhance the regulation of blood pressure and vascular reactivity, reduce serum cholesterol and triglycerides (60), and improve memory function in older adults (62). They also help to prevent obesity and diabetes by inhibiting specific enzymes (52). Phenolic compounds improve antioxidant activity by scavenging reactive oxygen and nitrogen molecules, chelating redox-active transition minerals, collaborating with other antioxidants, stimulating antioxidant enzymes and proteins, inhibiting pro-oxidant enzymes, and modulating transcription factors redox-sensitive (60). In Moreno-Montoro et al. (57), it was mentioned that catechin and gallic acid act as free radical scavengers, and that epicatechin has an antibacterial activity. In addition to their antioxidant capacity, gallic, caffeic, and chlorogenic acids act as venous dilators (49). Resveratrol plays a beneficial role in protection against various neoplasias, cardiovascular and neurodegenerative disorders, and viral infections as well as helps to retard body aging and reduces the incidence of heart and muscle diseases (56). Quercetin and its derivatives have shown anti-inflammation and anticarcinogenic properties when used in the treatment of some types of cancer (44).

During extended and intense exercises such as marathon and ultramarathon races, athletes exhibit severe physiological stress that appears as muscle microtrauma, oxidative stress, gastrointestinal dysfunction, or inflammation (63). According to (64), during long and extenuating physical exercises, oxidative stress is caused owing to a considerable increase of reactive oxygen species (ROS) promoting an imbalance in antioxidant capacity in the body, which leads to protein modification, lipid oxidation, DNA damage (65), inflammation (66), and chronic diseases including cancer and neurological and cardiovascular diseases (67). Grape and grape derivative products are a source of polyphenols (63) which are known of high antioxidant activities (68) and can be beneficial against oxidative damage (69). In addition, the carbohydrate content is useful for glycogen deposition and improvements of practice during long term exercises (64). Therefore, many researchers have studied the beneficial effect of grape juice related to improve performances during physical exercises. Table 2 shows some experimental studies carried out and their consequences, which have demonstrated that grape juice was able to improve performances and antioxidant activity, protect against oxidative damage, and reduce inflammation. The figure in the graphical abstract shows the contribution of grape juice in the development of an effective isotonic drink and its impact on athletes' performances.

**Table 2:** Different studies that demonstrate the impact of grape juice on performances during physical exercises.

**Grape juice**	**Experiment**	**Studied properties**	**Results**	**References**
Organic grape juice Bordeaux variety (*Vitis labrusca*)	10 adult male triathletesreceived organic grape juice (300 ml/day) for 20 days.	Glucose homeostasis Antioxidant status Cutaneous microcirculatory function	The results blood sample that was drawn before (baseline) and after 20 days showed that intake of grape juice improved glucose homeostasis, antioxidant capacity, and microvascular function.	(70)
Red grape juice khoshnam variety *Vitis vinifera*	30 male Wistar rats of Parkinson's disease were divided in groups and treated with grape juice, exercise, or grape juice associated with exercise for 30 days.	Neurodegenerative effect Parkinson's disease.	Rotations test demonstrated a reduction in the number of rotations in Parkinson's rats treated with grape juice and grape juice associated with exercise.	(71)
Organic purple grape juice Bordo variety *Vitis labrusca*	12 male Wistar rats were divided into 2 groups (nonexercised and exercised). Rats treated with organic purple grape juice were submitted to an exhaustive exercise bout.	Parameters of oxidative stress Protective impact	Evaluation of oxidative stress parameters showed that organic grape juice had capacity to protect different rat tissues against oxidative damage.	(72)
Purple grape juice Isabel, Bordeaux, and Concord varieties *Vitis labrusca*	28 recreational runners of both sexes were divided into 2 groups and received either grape juice or control beverage for 28 days.	Ergogenic impact Oxidative stress Inflammation Immune response Muscle injury	Results of time-to-exhaustion exercise, anaerobic threshold, and aerobic capacity test showed the ergogenic effect of grape juice in recreational runners by increasing performances, time-to-exhaustion, and antioxidant activity, and reducing inflammatory markers.	(66)
Purple grape juice, Isabel, Bordeaux and Concord varieties *Vitis labrusca*	2 groups of 28 healthy adults were assigned and received either grape juice or control beverage during intense and continuous physical exercise for 28 days.	Antioxidant activity, Lipid and glycemic profiles	Evaluation of the nutritional status, blood pressure, and blood collection upon receiving supplementation indicated that grape juice was a source of antioxidants improving antioxidant status and cardiometabolic profile.	(52)
Whole red grape juice American burgundy and Isabella grapes	A study was performed with 26 individuals with hypertension distributed into experimental group (supplementation with a daily dose of juice) and control groups (supplementation with a control drink) performed 2 sessions of aerobic exercise on a treadmill, separated by a 28-day period of supplementation with daily dose of grape juice or a control drink.	Impact on blood pressure of individuals with hypertension.	Measurement of blood pressure before, during, and after each exercise session showed that grape juice has a capacity to decrease blood pressure at rest and improve post-exercise hypotension in individuals with hypertension.	(73)
Purple grape juice Isabel, Bordeaux, and Concord varieties (*Vitis labrusca*)	14 recreational male runners performed two running tests to exhaustion after ingesting grape juice or a placebo drink (10 ml/kg/day).	Physical performance Oxidative stress Inflammation, and muscle damage	Ergogenic effect of grape juice by increasing time of run and improving of antioxidant activityin recreational runners.	(74)
Purple grape juice Bordeaux variety (*Vitis labrusca*)	12 male volleyball players participated in three different moments with match simulation: without beverage, grape juice, and placebo beverag for 14 days in a cross-over model.	Oxidative stress Inflammation Muscle damage	Grape juice promoted a reduction in protein oxidation, lipid peroxidation, and DNA damage.	(64)
Grape juice was Bordeaux variety (*Vitis labrusca*)	20 judo athletes wererandomized into 2 groups, and they consumed grape juice or placebo for 14 days in a crossover model	Oxidative stress Muscle fatigue parameters	Grape juice improved parameters of oxidative stress by reducing lipid and DNA damage.	(75)

### Sensory Characteristics of Grape Juice

Another interesting characteristic of grape juice is its great sensory characteristic. The phenolic compounds in grape juice are responsible for its sensory properties (color, flavor, and taste) (42). The phenolic acids affect the organoleptic properties of grape juice (49) and ensure a low pH value, which provides equilibrium between sweet and sour tastes (42). Accordingly, organoleptic properties are an important factor when choosing food products. Color is the most important characteristic used when choosing beverages (76). In addition, color is used by consumers as an indicator of juice quality (77), because there is a strong relationship between color and flavor, as consumers can be able to expect the flavor through the color of food products (78–80). Nowadays, replacement of synthetic dyes with natural colorant is a challenge for the food industry (76). The attractive orange, red, and purple colors and water solubility of anthocyanins allow their integration into aqueous food systems and use as natural colorants (81, 82). They also participate in multiple chemical reactions such as copigmentation and formation of polymeric pigments that contribute to color changes (77). For grape juice, cyanidin, peonidin, delphinidin, petunidin, and malvidin are the main anthocyanins responsible for the red color (83). Their stability is influenced by pH, temperature, oxygen, light, presence of ascorbic acid and metal ions, and high concentration of sugar (42, 82, 84). On the other hand, consumption of anthocyanins promotes health benefits by reducing the risk of cancer, inflammation, neuronal and cardiovascular diseases, diabetes, obesity, and cognitive function disorders (85) owing to their antioxidant anti-cardiovascular, anticancer, anti-inflammatory, anti-thrombotic, anti-ulcer, anti-allergenic, and anti-coagulant activities as well their immunomodulatory, vasodilatory, and analgesic activities (86).

## Conclusion

Since creation of new, natural, functional, and healthy products based on fruit juices is a challenge for the food industry, grape juice represents a suitable alternative for the development of a new isotonic drink. An isotonic beverage that is designed to rehydrate, replenish electrolytes, and promote energy could be more effective when enriched with grape juice by diluting the sugar content of grape juice to 40–50 g/L to obtain a beverage with beneficial health properties. Besides rehydration, isotonic drinks have more benefits in terms of antioxidant activity because of the phenolic content, which acts against oxidative stress related to intense sports activities. In addition, grape juice is a natural source of sugars, which leads to avoidance of adding sweeteners, and plays an important role in glycogen compensation. Moreover, attractive sensory characteristics, mainly color which is provided by anthocyanin content, have a great contribution to make the drink more natural and help to dispense the use of synthetic dyes. Finally, developing new and natural isotonic beverages based on grape juice with antioxidant capacity and interesting sensory properties will be a novel product in the field of healthy beverages.

## Author Contributions

YB: investigation. YB, CV, CG, and AM: writing—original draft and review and editing. AM and CV: visualization. YB, CV, and AM: validation. AM and CG: conceptualization. All authors have read and agreed to the published version of the manuscript.

## Conflict of Interest

The authors declare that the research was conducted in the absence of any commercial or financial relationships that could be construed as a potential conflict of interest.

## Publisher's Note

All claims expressed in this article are solely those of the authors and do not necessarily represent those of their affiliated organizations, or those of the publisher, the editors and the reviewers. Any product that may be evaluated in this article, or claim that may be made by its manufacturer, is not guaranteed or endorsed by the publisher.
